# Editorial: Recent Evolutions and Perspectives in Olympic Winter Sports Performance: To PyeongChang and Beyond…

**DOI:** 10.3389/fphys.2019.00481

**Published:** 2019-04-24

**Authors:** Gianluca Vernillo, Nicolas Coulmy, Grégoire P. Millet

**Affiliations:** ^1^Department of Biomedical Sciences for Health, University of Milan, Milan, Italy; ^2^French Ski Federation, Annecy, France; ^3^Faculty of Biology and Medicine, Institute of Sport Sciences, University of Lausanne, Lausanne, Switzerland

**Keywords:** athlete, olympic games, performance, training, winter sports

## Introduction

The XXIII Olympic Winter Games were hosted in PyeongChang (South Korea) from the 9th to the 25th of February 2018. The edition included 102 events over 15 disciplines in seven sports. A total of 2,914 athletes from 92 countries competed, including several emerging countries in the winter sports panorama such as Kosovo, Eritrea, Nigeria, and Singapore.

An important goal for most athlete-centered research is to be translated into practice, it is used to inform the development of improved athlete preparation and/or performance (Coutts, 2017). The result of this process is commonly defined as evidence-based practice. Though the efficacy of translating athlete-centered research to practice is still debated (for reasons beyond the scope of this editorial; Bishop, 2008; Coutts, 2017), this Research Topic sought to provide a platform for papers on Olympic winter sports, and set out a comprehensive framework of the different attributes that should be addressed within Olympic winter sports performances. The aim was therefore, on one hand: to better understand factors determining athlete performance, recent changes, the evolution in training content and methods, the improvement in technology, as well as the occurrence of injury and illness across the different Olympic winter sports. On the other hand, we wanted to provide the opportunity not only to translate research to practice, but more importantly to guide the practice of Olympic winter sports, with the ultimate goal of enhancing the performance, improving technological means and equipment, and/or reducing injury risks. Therefore, we focused on issues related to how winter sports athletes meet the acute physiological and biomechanical demands of their winter discipline, and also on the factors that govern some of the long-terms adaptations to exercise training. To explore this, we welcomed the submission of original research, review, perspective articles, and case studies on elite Olympic winter sport athletes, which specifically considered the scope, and impact of their findings in the broader context of Olympic winter sports performance.

## Topic Content

A total of 18 papers have been accepted (10 original research papers, four perspectives, two case reports, one review, and a brief research report), written by in total 69 contributing authors from 40 different laboratories (including three winter sports federations) and from 11 countries. Overall, the papers were all directed toward a better understanding of physiological, biomechanical, and training factors related to different Olympic winter sports disciplines: cross-country skiing, alpine skiing, biathlon, Nordic combined, speed skating, snowboarding, and ski-cross.

## Cross-Country Skiing

Cross-country skiing is one of the most physiologically demanding sport among the endurance disciplines (Holmberg, 2015). Indeed, it poses significant physiological challenges by means of a combined upper- and lower-body effort. Arm and leg muscles exhibit differences in the way that arm muscles present a lower fat oxidation together with being less oxidative and less capable of extracting O_2_ (Calbet et al., 2005). Ørtenblad et al. compared the equally trained limb muscles of elite cross-country skiers. They observed that despite the mitochondrial volume percentage and the number of capillaries per fiber area resulted similar in the arms and legs, arms presented more MHC-2 fibers, and larger type 2A fibers (likely due to the demands of the modern cross-country skiing toward rapid generation of large forces during short contact periods). Conversely, lipid metabolism was higher in the leg muscle. These findings can open new perspectives in terms of training regimes applied to the modern cross-country skiing training. However, besides being one of the most physiologically demanding sports, cross-country skiing also involves highly complex biomechanics (Smith, 1990). Pellegrini et al. in their perspective article argued that, throughout modern sport history, cross-country skiing is probably the sport that has evolved the most from a biomechanical and technical point of view. Interestingly, the authors reasoned that despite the enormous changes in recent decades, there still is room for further developments. These improvements include the use of inertial sensors to monitor a skier's speed, motion, and technique continuously and non-invasively. On that matter, Gløersen et al. developed a procedure for estimating the propulsive power generated during roller-skiing using small non-intrusive sensors. The authors observed that the error in the estimation of the propulsive power increased with skiing speed. On the other hand, the propulsive power generated decreased approximately linearly as speed increased. An accurate measurement/estimation of the propulsive power throughout different cross-country ski races is therefore required to improve the understanding of the specific work requirements during cross-country skiing races of different distance. This aspect seems to be of paramount important in cross-country skiing, since Marsland et al. observed differences in the macro-kinematic characteristics utilized during either sprint or distance events. Further, Zoppirolli et al. observed attenuated generation of double-poling forces during a fatiguing 58-km cross-country skiing marathon that could be overcome by maintaining optimal elbow and ankle kinematics and an effective forward lean during the propulsive phase. However, the characterization of cross-country skiing was not merely met in this Research Topic by means of physiological and biomechanical reports. Indeed, from a training perspective, Schmitt et al. highlighted that in elite cross-country skiing athletes the training intensity distribution was shifted toward ~82% of low intensity training (defined as intensities below the first ventilatory threshold). An important insight has also been provided by Solli and Sandbakk's case study, which described the training characteristics during pregnancy and postpartum in the world's most successful cross-country skier. Notably, the topic of women competing at the elite level while pregnant is an unquestionable important area of research and there is a paucity of controlled physiological studies (Wagner, 2012).

## Alpine Skiing

The nature of the alpine skiing training toward performance is multifactorial and based on an interaction between intrinsic (e.g., physiological and psychological) and extrinsic (e.g., environment and materials) characteristics (Impellizzeri et al., 2009). An interesting perspective article covering this aspect has been done by Gilgien et al. The authors, by means of a survey administered to the coaching staff, described how Olympic athletes from Germany, Norway, Sweden, and Switzerland prepared the XXIII Olympic Winter Games in PyeongChang (South Korea) in 2018. The survey items were based on the athletes' typical exercise programs with respect to physical conditioning, ski training, and periodization. Notably, the authors highlighted how the training periodization does not typically follow a traditional annual cycle; rather it is influenced by the availability of good in-snow training conditions. Two distinct characteristics of the alpine skiing training were also highlighted: the importance of strength training (in its different forms) and the careful attention given to training in hypoxic environments. The interaction between hypoxia and strength has been the object of Alhammoud et al.'s study, who explored the effects of acute hypoxia on maximal (as well as explosive) torque and fatigability in the knee extensors of elite alpine skiers. This highlighted the importance of considering explosive strength measurements in the alpine skiing neuromuscular screening tests and prevention programs. The training management between in- and off-snow period is a challenging aspect of alpine skiing (Turnbull et al., 2009). In order to address this issue, Stöggl et al. explored the feasibility of using a dryland ski ergometer as a training tool. Results showed that the high intensity training protocols administered with the ski ergometer were able to achieve a high level of cardiorespiratory and metabolic responses. This highlights the feasibility of using a ski ergometer during the off-snow period in alpine skiing to effectively train the endurance component. However, alpine skiing is a complex winter sport discipline and requires not only high levels of physical capacities (Andersen and Montgomery, 1988; Raschner et al., 2017) but also complex biomechanical features. This aspect has been the object of three papers accepted in this Research Topic. Supej and Holmberg provided an update on the biomechanics of alpine skiing, highlighting the importance of the inclusion of measurement technologies in order to optimize the coaches' work. Some of these technologies have been explored by Fasel et al. and Meyer and Borrani. Fasel et al. presented and validated a method to correct velocity and position drift for inertial sensor-based measurements. Whereas, Meyer and Borrani explored the link between the antenna trajectory of global navigation satellite system and the alpine skiers' center of mass.

## Biathlon

Biathlon combines rifle marksmanship and cross-country skiing while carrying a rifle. It presents similar physiological requirements to those observed for cross-country skiing (Holmberg, 2015) together with precise fine motor control for fast and accurate shooting (Vickers and Williams, 2007). The extensive perspective article by Laaksonen et al. covered both training and technical (i.e., skiing and shooting) aspects of modern biathlon, highlighting the needs to concurrently optimize physiological and performance capacities of cross-country skiing techniques with shooting.

## Nordic Combined

As described by Rasdal et al. (2017), Nordic combined is an Olympic winter sport where the athletes have to compete in both a ski-jumping event and a cross-country skiing race on the same day. Despite existing similarities, cross-country skiers, and Nordic combined athletes possess different characteristics and training contents (Sandbakk et al., 2016). This was also interestingly observed by Schmitt et al. who presented differences in the training intensity distribution between cross-country skiers and Nordic combined athletes. If cross-country skiers presented a training intensity distribution shifted toward low intensity training regimes (see above), Nordic combined athletes presented a lower low intensity volume (~51%) but higher strength and speed training (~39%). In support of this, Rasdal et al. presented the case study of a Nordic combined champion, highlighting his training, and technical and physiological development during the last four seasons preceding the XXIII Olympic Winter Games in PyeongChang (South Korea) in 2018.

## Speed Skating

Speed skating is a peculiar sport in which athletes adopt a crouched position and push-off sideward in order to move forward (Noordhof et al., 2014). The characteristics of speed skating contribute to impede blood flow and exacerbate deoxygenation in the lower limbs that is remarkably greater for short- vs. long-track speed skating (Hettinga et al., 2016). Richard and Billaut explored whether or not combining preconditioning strategies could modify muscular oxygenation and improve speed skating performance. Results showed that a preconditioning strategy of combined remote ischemic preconditioning and inspiratory muscle warm-up did not significantly impact the 600-m speed skating performance in elite skaters. This highlights the need for further studies to clearly identify positive strategies to impact speed skating performance, particularly by reducing skeletal muscle deoxygenation.

## Snowboarding

Similar to alpine skiing, snowboarding requires high technical skills, leg strength, dynamic ability as well as aerobic and anaerobic capacity (Vernillo et al., 2016a,b, 2017). Though snowboarding has grown in popularity as an Olympic winter sport, only a small number of studies analyzing the physiological and performance characteristics, as well as the requirements of snowboarding, have been published thus far. The review by Vernillo et al. explores the current literature providing insights into the physiological and physical characteristics of snowboarding performance. It revealed the need to improve our current understanding of snowboarding, as well as its physiological and performance profiles, in order to more effectively train snowboarding athletes.

## Ski-Cross

Ski-cross is a type of skiing competition where four skiers are required to maneuver inside a course characterized by multiple obstacles (e.g., banks and jumps). A distinctive feature of this discipline is that athletes can take advantage of the so-called slipstreaming (or drafting) to catch up with the leading athlete. If slipstreaming is a well-studied strategy in those sports where the preservation of muscle power is a fundamental requirement (e.g., cycling, speed skating, and running), then how it affects gravity-powered sports is less investigated. Fuss derived a strategy for slipstreaming in ski-cross by means of a glide model. Results showed that the glide model could be used to test the design of the slope track, precisely identifying the dimensions of the terrain features, and thereby deriving the skiers' speed as well as identifying the critical section of the course.

## Conclusion

The scientific basis of Olympic winter sports, as well as the studies of how athletes cope with the specific physiological and biomechanical requirements of their discipline, is of paramount importance and interest for exercise physiologists, sport scientists, and particularly coaches. These studies are necessary in order to improve the current understanding of Olympic winter sports performance. The continued evolution of Olympic winter sports is contributing to an enormous accumulation of knowledge, evidence, and relevant training technologies. In this context, the 18 papers that constitute this Research Topic have contributed to our better understanding of the requirements of different Olympic winter sports. The field of study is enormous and touches a considerable number and range of issues. We hope this Research Topic will contribute to the stimulation of further research in all the aspects highlighted within it. The ultimate goal being to improve all Olympic winter sports performances. With the recent implementation by the International Olympic Committee of the Youth Olympic Games, further research is also required to better analyse the practice of winter sports by young athletes at the elite level. Questions about the optimal increase in training load and the specificity of the training content at different age-stages for reducing health and risk of injury are paramount and yet minimally investigated. The next step might come from the “Youth and Winter Sports” congress being held from the 7th to the 8th of January 2020 in Lausanne (Switzerland) prior the next winter Youth Olympic Games, also hosted in Lausanne from the 9th to the 22nd of January 2020.

## Author Contributions

All authors listed have made a substantial, direct and intellectual contribution to the work, and approved it for publication.

### Conflict of Interest Statement

The authors declare that the research was conducted in the absence of any commercial or financial relationships that could be construed as a potential conflict of interest.

## References

[B1] Andersen R. E. Montgomery D. L. (1988). Physiology of alpine skiing. Sports Med. 6, 210–221. 10.2165/00007256-198806040-00003 3067309

[B2] Bishop D. (2008). An applied research model for the sport sciences. Sports Med. 38, 253–263. 10.2165/00007256-200838030-00005 18278985

[B3] Calbet J. A. Holmberg H. C. Rosdahl H. van Hall G. Jensen-Urstad M. Saltin B. (2005). Why do arms extract less oxygen than legs during exercise? Am. J. Physiol. Regul. Integr. Comp. Physiol. 289, R1448–R1458. 10.1152/ajpregu.00824.2004 15919729

[B4] Coutts A. J. (2017). Challenges in developing evidence-based practice in high-performance sport. Int. J. Sports Physiol. Perform. 12, 717–718. 10.1123/IJSPP.2017-0455 28832264

[B5] Hettinga F. J. Konings M. J. Cooper C. E. (2016). Differences in muscle oxygenation, perceived fatigue and recovery between long-track and short-track speed skating. Front. Physiol. 7:619. 10.3389/fphys.2016.00619 28018244 PMC5156719

[B6] Holmberg H. C. (2015). The elite cross-country skier provides unique insights into human exercise physiology. Scand. J. Med. Sci. Sports. 25 (Suppl. 4), 100–109. 10.1111/sms.12601 26589123

[B7] Impellizzeri F. M. Rampinini E. Freschi M. Maffiuletti N. A. Bizzini M. Mognoni P. (2009). Identification of the physical characteristics that discriminate between competitive levels and specialities of alpine skiers, in Science and Skiing IV, eds Muller E. Lindinger S. Stöggl T. (Aachen: Meyer & Meyer Sport), 272–280.

[B8] Noordhof D. A. Foster C. Hoozemans M. J. de Koning J. J. (2014). The association between changes in speed skating technique and changes in skating velocity. Int. J. Sports Physiol. Perform. 9, 68–76. 10.1123/ijspp.2012-0131 23319446

[B9] Raschner C. Hildebrandt C. Mohr J. Muller L. (2017). Sex differences in balance among alpine ski racers: cross-sectional age comparisons. Percept. Mot. Skills. 124, 1134–1150. 10.1177/0031512517730730 28901201

[B10] Rasdal V. Fudel R. Kocbach J. Moen F. Ettema G. Sandbakk O. (2017). Association between laboratory capacities and world-cup performance in Nordic combined. PLoS ONE 12:e0180388. 10.1371/journal.pone.0180388 28662163 PMC5491221

[B11] Sandbakk O. Rasdal V. Braten S. Moen F. Ettema G. (2016). How do world-class nordic combined athletes differ from specialized cross-country skiers and ski jumpers in sport-specific capacity and training characteristics? Int. J. Sports Physiol. Perform. 11, 899–906. 10.1123/ijspp.2015-0285 26791774

[B12] Smith G. A. (1990). Biomechanics of crosscountry skiing. Sports Med. 9, 273–285. 10.2165/00007256-199009050-00003 2188330

[B13] Turnbull J. R. Kilding A. E. Keogh J. W. (2009). Physiology of alpine skiing. Scand. J. Med. Sci. Sports 19, 146–155. 10.1111/j.1600-0838.2009.00901.x 19335589

[B14] Vernillo G. Pisoni C. Sconfienza L. M. Thiebat G. Longo S. (2017). Changes in muscle architecture of vastus lateralis muscle after an alpine snowboarding race. J. Strength Cond. Res. 31, 254–259. 10.1519/JSC.0000000000001469 27135473

[B15] Vernillo G. Pisoni C. Thiebat G. (2016a). Physiological characteristics of elite snowboarders. J. Sports Med. Phys. Fitness 56, 527–533. 25924566

[B16] Vernillo G. Pisoni C. Thiebat G. (2016b). Strength asymmetry between front and rear leg in elite snowboard athletes. Clin. J. Sport Med. 26, 83–85. 10.1097/JSM.0000000000000194 25881565

[B17] Vickers J. N. Williams A. M. (2007). Performing under pressure: the effects of physiological arousal, cognitive anxiety, and gaze control in biathlon. J Mot. Behav. 39, 381–394. 10.3200/JMBR.39.5.381-394 17827115

[B18] Wagner P. D. (2012). On completing a marathon and giving birth the same day. J. Appl. Physiol. 113:987. 10.1152/japplphysiol.00659.2012 22678959

